# Rebound effect explains the divergence in survival after 5 days in a controlled trial on vitamin C for COVID-19 patients

**DOI:** 10.3389/fmed.2024.1391346

**Published:** 2024-05-21

**Authors:** Harri Hemilä, Elizabeth Chalker

**Affiliations:** ^1^Department of Public Health, University of Helsinki, Helsinki, Finland; ^2^National Centre for Epidemiology and Population Health, Australian National University, Canberra, ACT, Australia

**Keywords:** ascorbic acid, intensive care, mortality, SARS-CoV-2, scurvy, sepsis, time factors, vitamins

## 1 Introduction

In empirical studies, sudden termination of high-dose vitamin C has been shown to cause a fall in vitamin C levels to levels below those recorded prior to the high-dose administration (1–4). This phenomenon is called the rebound effect. These studies are old and the vitamin C doses were much less than the dose in the study analyzed in this paper. Unfortunately, there do not seem to be more recent studies, but it seems highly likely that the rebound effect will be observed more strongly with higher doses of vitamin C and with critically ill patients. In certain contexts, the abrupt termination of high-dose vitamin C may dramatically decrease plasma levels and lead to harmful effects on health. The rebound effect was also observed in a guinea pig study, which reported increased mortality after the termination of very high vitamin C doses (5).

Previously we showed that the abrupt termination of 4-day intravenous vitamin C in the LOVIT trial with 862 sepsis patients explained the harm observed in the participants randomized to the vitamin C group (6, 7). After randomization, patients in the vitamin C group were administered vitamin C with a dose of 50 mg/kg body weight every 6 h (16 g/day for an 80 kg person). There was no difference in survival between the trial groups during the 4-day vitamin C administration. However, during days 5 to 7 there was significantly elevated mortality in the vitamin C group with RR = 2.28 (95% CI: 1.24–4.2; vitamin C 33 vs. placebo 15 deaths) (7). After this sharp increase in mortality immediately after the termination of vitamin C, the difference in survival curves leveled off (6, 7). This pattern is consistent with the rebound effect as the cause of the short-term difference between the trial groups.

The CITRIS-ALI trial administered intravenous vitamin C with the same dosage of 50 mg/kg every 6 h for up to 4 days to sepsis patients (8, 9). We showed that there was a statistically significant change in the relationship between the survival curves immediately after the termination of vitamin C in this trial also (10). Vitamin C was significantly beneficial during the 4-day administration, but the benefit sharply disappeared when vitamin C administration was ceased (8, 10). Thus, in these two trials the sudden termination of the 4-day intravenous vitamin C administration caused a substantial short-term change in mortality.

Meta-analyses of large numbers of controlled trials have shown that vitamin C has beneficial effects on unspecified respiratory virus infections (11, 12), and there is also justification that the benefits extend to SARS-CoV-2 infection (13). The COVID A to Z trial of COVID-19 outpatients administered 8 g/day of vitamin C for 10 days (14), and vitamin C increased the rate of recovery by 70% (95% CI 6.8% to 170%, *P* = 0.025) (15, 16).

A recent study examined the effect of 4-day intravenous vitamin C for COVID-19 patients with a dose of 50 mg/kg every 6 h (17). The patients were also allocated to two groups by the severity of disease: a) patients who were critically ill and b) those who were not critically ill. In the published survival curves of the critically ill patients, visually there is no difference between the vitamin C and control groups during the 4-day vitamin C administration, but there is dramatic divergence soon after the termination of vitamin C. In this paper we analyze the survival curves.

## 2 Analysis of the survival curves in the trial on vitamin C for COVID-19 patients

The methods of the study on vitamin C and COVID-19 patients have been described previously by Adhikari et al. (17). In brief, two prospectively harmonized randomized clinical trials enrolled critically ill patients receiving organ support in ICU and patients who were not critically ill. The primary outcome was a composite of organ support-free days defined as days alive and free of respiratory and cardiovascular organ support in intensive care up to day 21, and survival to hospital discharge. The range is from −1 (in-hospital death) to 22 for those who survived without needing organ support. The results of the two separate trials were pooled at the end, with 1493 patients assigned to vitamin C and 1097 assigned to the control group.

**Figure 1:**
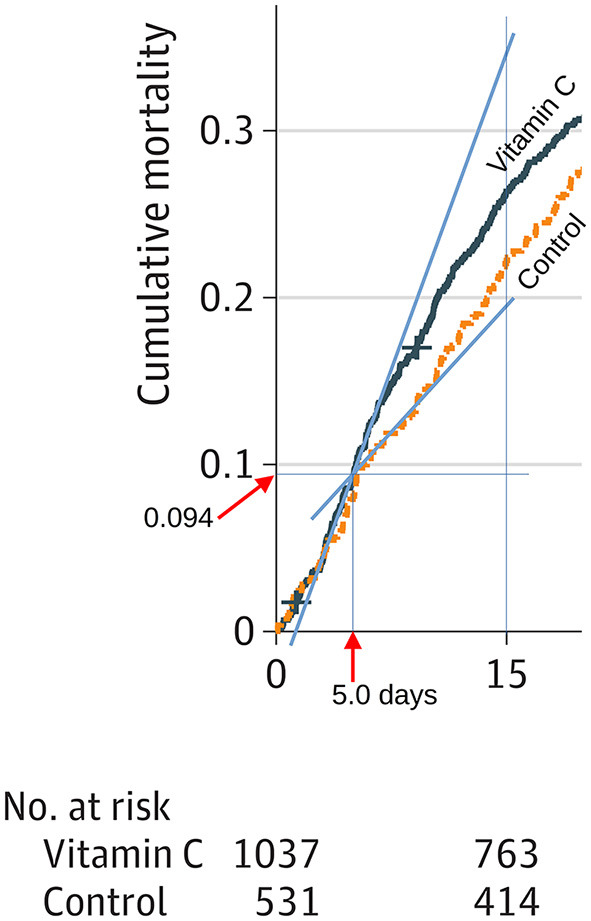
Divergence in the vitamin C and control group survival curves in the trial on vitamin C for COVID-19 patients. We drew the blue lines to estimate the slopes immediately after the termination of vitamin C administration. We measured the distances with a graphics program and determined that the divergence starts at ~5.0 days. The cumulative mortality at the start of the divergence is 0.094. Addition of a separate vitamin C effect for the period 5 to 7.5 days improves the Poisson regression model by χ^2^ = 6.7 (P = 0.010). See the calculations in the Supplementary material.

In their Figure 2C, Adhikari et al. (17) published the survival curves of the critically ill vitamin C and control patients. The follow-up time in the published figure is 90 days which makes it difficult for a reader to detect the substantial divergence between the survival curves during the early follow-up period. Our Figure 1 shows an enlargement of the early period.

We drew guide lines to estimate the time point of the divergence between the two survival curves and determined that to be 5.0 days. The survival proportion at this time point corresponds to a cumulative mortality level of 0.094, which corresponds to 98 patients in the vitamin C group, and 50 participants in the control group. Using these mortality estimates, we calculated the number of patients at risk at day 5: vitamin C 939 and control 481; see the Supplementary material.

Given the published number of patients remaining at day 15 [vitamin C 763; control 414 (17)], we calculated that between days 5 and 15, there were 176 deaths in the vitamin C group and 67 deaths in the control group. These data indicate that in the time range from 5 to 15 days, those in the vitamin C group had 1.35 times the risk of dying than those in the control group (RR = 1.35; 95% CI 1.04–1.74; *P* = 0.026). This increased mortality occurred directly after the termination of the 4-day intravenous vitamin C.

However, the RR = 1.35 is an underestimate of the immediate harm after the sudden termination of vitamin C. After day 10, the survival curves of the vitamin C and control groups appear equidistant, indicating that the main harm from the abrupt cessation of vitamin C occurred between day 5 and 10; see Supplementary Figures. Therefore, we also estimated the increased risk in mortality in the vitamin C group from the slopes of the blue lines shown in Figure 1. This approach yields RR = 2.5 as the estimate for the increased risk immediately after the termination of vitamin C administration.

As a third approach, we measured the heights of the survival curves at 7.5 days when the divergence started to level off. We calculated that in the time range 5 to 7.5 days there were 56 deaths in the vitamin C group and 13 in the control group, which yields the increased mortality in the vitamin C group soon after the termination of vitamin C as RR = 2.21 (95% CI 1.22–4.0; *P* = 0.006). This method covers a longer time range than the calculation from the slopes at the intersection, yet the point estimates are consistent and a confidence interval for the estimate can be calculated. Furthermore, addition of a separate vitamin C effect for the period 5 to 7.5 days improves the Poisson regression model by χ^2^ = 6.7 which corresponds to *P* = 0.010; see the Supplementary material.

Adhikari et al. (17) reported the effect of 4-day intravenous vitamin C administration on the mortality of COVID-19 patients who were not critically ill in their Figure 3C, and on the primary outcome in Figure 3A. There are no meaningful differences between the groups in either figure.

## 3 Discussion

In our earlier reanalysis of the LOVIT trial on sepsis patients we found that for the 3 days immediately following the abrupt termination of the 4-day vitamin C administration, mortality in the vitamin C group dramatically increased to RR = 2.28, after which the difference between the survival curves leveled off (6, 7). In the current analysis of the identical 4-day vitamin C administration to critically ill COVID-19 patients, we calculated that immediately after the termination of vitamin C the risk of mortality in the vitamin C group increased to RR = 2.21 which is very close to the estimate for sepsis patients (7). The harm from sudden termination of high-dose vitamin C can be explained by the rebound effect (1–5, 7). Adhikari et al. (17) neglected to mention the previous report (7) even though the vitamin C dosage was identical.

In their introduction to the report, Adhikari et al. (17) stated that two clinical trials (18, 19) had not demonstrated any benefit from vitamin C to sepsis patients. However, Adhikari did not refer to a Korean cohort study which found that the combination of vitamin C and hydrocortisone behaved differently from vitamin C alone, in that vitamin C alone appeared beneficial but the combination did not (20). Although we need to be cautious about treatment effects estimated in observational studies, the Korean findings question the validity of the vitamin C and hydrocortisone combination trials to which Adhikari referred (18, 19). To truly examine the effects of vitamin C, the only difference between the treatment groups should be vitamin C alone, and not combinations with other substances (21).

Furthermore, Adhikari et al. (17) did not justify the choice of 4-day vitamin C administration. Vitamin C levels are low in critically ill patients, which may reflect increased consumption of the vitamin in the system (6, 22). In Adhikari's Figure 2A, about 70% of patients had “organ support-free days” for 17 days or less. Given the 21-day follow-up, this indicates that these patients had organ support for 4 days or more. Thus, about 70% of patients had ongoing organ support after the abrupt termination of the 4-day vitamin C. When there is ongoing increased consumption of vitamin C in the system, the rebound effect may be particularly pronounced, yet this issue was not considered (17). Adhikari's results with the short 4-day administration do not contradict the findings of meta-analyses in which vitamin C shortened ICU stay by 7.8% (22) and the duration of mechanical ventilation by 25% (23).

Although Adhikari et al. (17) studied COVID-19 patients, they did not refer to the COVID A to Z trial (14), which found a 70% increase in the recovery rate of outpatient cases of COVID-19 patients when administering 8 g/day vitamin C for 10 days (15). Furthermore, the quantile treatment effect approach indicated that there was no evidence of benefit for patients who suffered from COVID-19 for less than a week, whereas 4-week symptoms were shortened by about 2 weeks (15, 16).

Adhikari et al. (17) did not consider potential explanations for the conflicting findings: benefit in the COVID A to Z trial but lack of benefit in their study with not-critically ill COVID-19 patients. One potential explanation is the short duration of treatment in their trial (4 days vs. 10 days in the COVID A to Z trial). A second potential explanation is the chosen primary outcome. Adhikari et al. (17) decided on a primary outcome focused on the duration of organ support in the ICU, but such an outcome can be insensitive for patients who are less ill. In fact, the median for the composite primary outcome was 22 days (i.e., maximum) in both groups of the patients who were not critically ill. In contrast, in the COVID A to Z trial, the primary outcome was the duration of symptoms which is a much more sensitive measure.

Cases of scurvy have been reported in critically ill patients and there are numerous reports of patients suffering from scurvy even in Western hospitals (24–37). A few critically ill patients have died of scurvy (38–42). We are concerned that vitamin C may be widely discouraged from use in critically ill patients on the basis of harm caused by the abrupt termination of vitamin C, without any indication that ongoing vitamin C administration is harmful (6, 7, 17). Very low vitamin C levels have been reported in COVID-19 patients (13, 43, 44). Furthermore, scurvy and COVID-19 can co-exist (45).

The efficacy of vitamin C for patients with COVID-19 remains uncertain. However, the Adhikari et al. (17) trial should not be considered as a counterargument to giving vitamin C to COVID-19 patients. Instead, all three trials that terminated 4-day vitamin C administration abruptly (6, 8, 9, 17), provide evidence that vitamin C should not be abruptly stopped if the patients are still critically ill. Given that scurvy is a serious and potentially life-threatening disease, it is not ethical to withhold vitamin C from patients who have very low vitamin C levels (7, 36). Nevertheless, further research of the rebound effect is needed to monitor plasma vitamin C levels in contexts that do not compromise the safety of patients.

## Author contributions

HH: Conceptualization, Methodology, Visualization, Writing—original draft, Writing—review & editing. EC: Data curation, Methodology, Validation, Writing—review & editing.
