# Supplementary material for: Rebound effect explains the divergence in survival after 5 days in a controlled trial on vitamin C for COVID-19 patients
Source: Front Med (Lausanne). 2024 May 21;11:1391346. doi: 10.3389/fmed.2024.1391346 (PMC11151746; doi:10.3389/fmed.2024.1391346)
Supplement: Supplementary file 1 [file Data_Sheet_1.pdf]

Rebound effect explains the divergence in survival after 5 days in a controlled trial on vitamin C for COVID-19 patients

Harri Hemilä and Elizabeth Chalker

Supplementary file
2024-5-14

Frontiers in Medicine:
https://www.frontiersin.org/articles/10.3389/fmed.2024.1391346/full
https://www.frontiersin.org/articles/10.3389/fmed.2024.1391346/full#supplementary-material

This supplement contains the figures that were analyzed in the paper.

Figure 2C of Adhikari et al. [17] was extracted from the paper and used in our analyses.

17. LOVIT-COVID Investigators, on behalf of the Canadian Critical Care Trials Group, and the REMAP-CAP Investigators; Adhikari NKJ et al.
Intravenous vitamin C for patients hospitalized with COVID-19: two harmonized randomized clinical trials.
JAMA. 2023;330(18):1745-1759.
https://doi.org/10.1001/jama.2023.21407
https://www.ncbi.nlm.nih.gov/pmc/articles/PMC10600726

Adhikari et al. published the survival curves for vitamin C and control groups over 90 days. We show the period up to 15 days, since the termination of vitamin C administration took place after 4 days and therefore this range is most relevant when considering potential effects of vitamin C and its abrupt termination. While there is evidence that the survival curves differ between days 5 to 7.5, there is no evidence that the survival curves differ between days 15 and 90 days with 95%CI for the RR: 0.73 to 1.18, see page 7. Thus, publishing the Figure 2C with the time range up to 90 days [17] camouflages the divergence at the early part of the curves and thereby misleads the readers.

Contents **Page**

Figure A (extracted from [17])	2
Figure B (extracted from [17])	3
Figure C (extracted from [17])	4
Calculation of the RR to compare vitamin C and control groups:	
Time range 5 to 15 days (compare page 2)	5
Time range 5 to 7.5 days (compare page 4)	6
Time range 15 to 90 days of Figure 2C in [17]	7
Improvement in Poisson regression by adding vitamin C effect on day range 5.0 to 7.5 days	8

In **Figure A** (extracted from [17]) we drew blue guide lines to show the divergence between the two groups. Using a graphics program, we measured the positions as pixels from the upper left corner and transformed the measurements to the units of the axis. Our Figures A to C can be extracted from this PDF file. We determined that the survival curves started to diverge at 5.0 days and the blue lines crossed at a cumulative proportion of mortality of 0.094, which corresponds to 98 deaths in the vitamin C group and 50 deaths in the control group. See the calculation below the Figure.

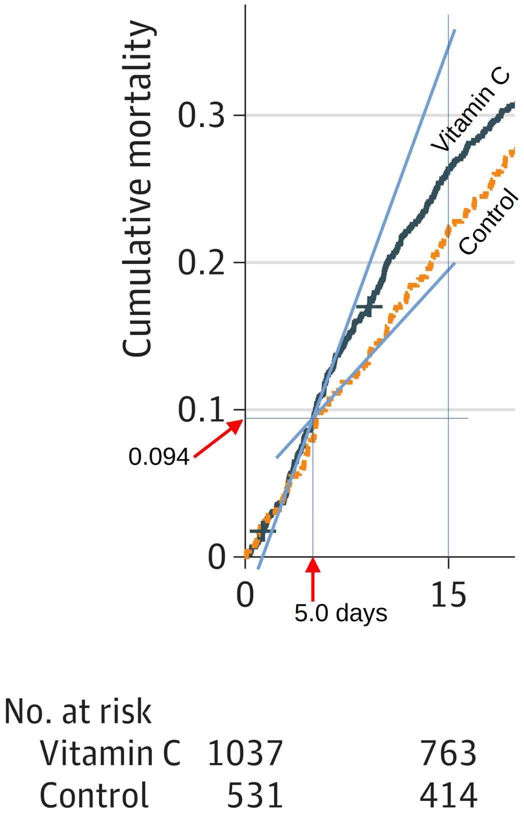

**Horizontal (days)**				Start of divergence
		Pixels	Pixel difference	Days
	15	1764	754	**15.00**
**blue**		1261	251	**4.99**
	0.0	1010		

**Vertical (cumulative mortality)**				Mortality at divergence
		Pixels	Pixel difference	
	0.3	410	1642	**0.3000**
**blue**		1537	515	**0.0941**
	0.0	2052		

	**Vitamin C**	**Control**
Time 0	1037	531
At 4.8 days	**97.6**	**50.0**
**Rounded to**	**98**	**50**
**At risk at day 5**	939	481
**Alive at day 15**	763	414
**Died between day 5 and day 15**	**176**	**67**
**Rate of death between day 5 and 15**	0.19	0.14
**RR for death between days 5 and 15**	**1.346**	

**Slopes**	Pixels	Pixel difference	RR
Vitamin C	159	1378	**2.51**
Control	987	550	
0.0	1537		

The pixel levels are measured at day 15

The "0-level" indicates the level at the blue horizontal line (mortality level 0.0941)

In **Figure B** (extracted from [17]) we drew parallel blue guide lines to show that the survival curves of the two groups are parallel after the short-term divergence. The vertical line indicates the approximate start of the parallel region.
We determined that the parallelism starts at about 10 days, see calculation below the Figure.

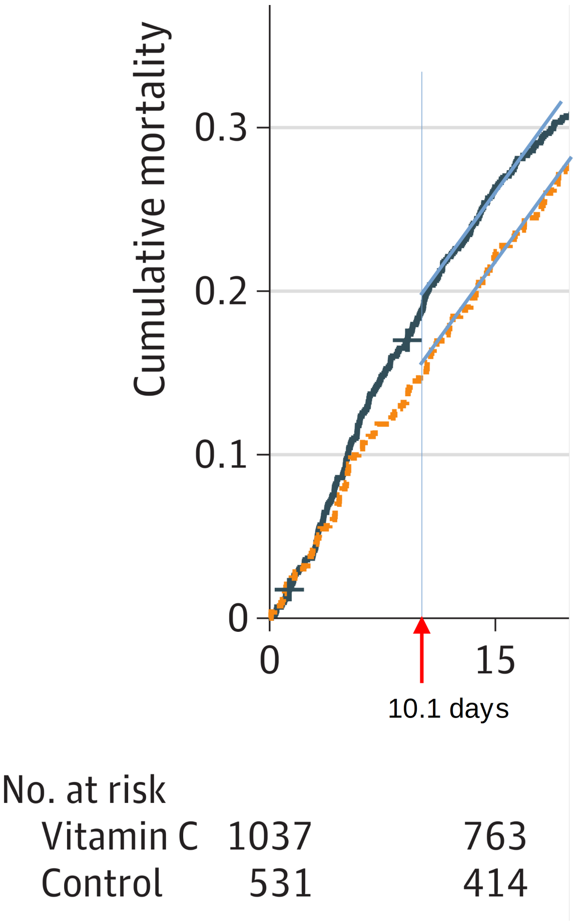

Figure 2C_B: Estimation of the start of the parallellism

**Horizontal (Days)**		Start of parallelism		
				Days
		Pixels	Pixel difference	
	15	1764	754	**15.0**
**blue**		1518	508	**10.1**
	0.0	1010		

In **Figure C** (extracted from [17]) we drew a blue guide line to estimate the levels of the survival curves when the divergence started to level off. The blue line is located at 7.5 days, see calculation of the survival curve levels below the Figure.

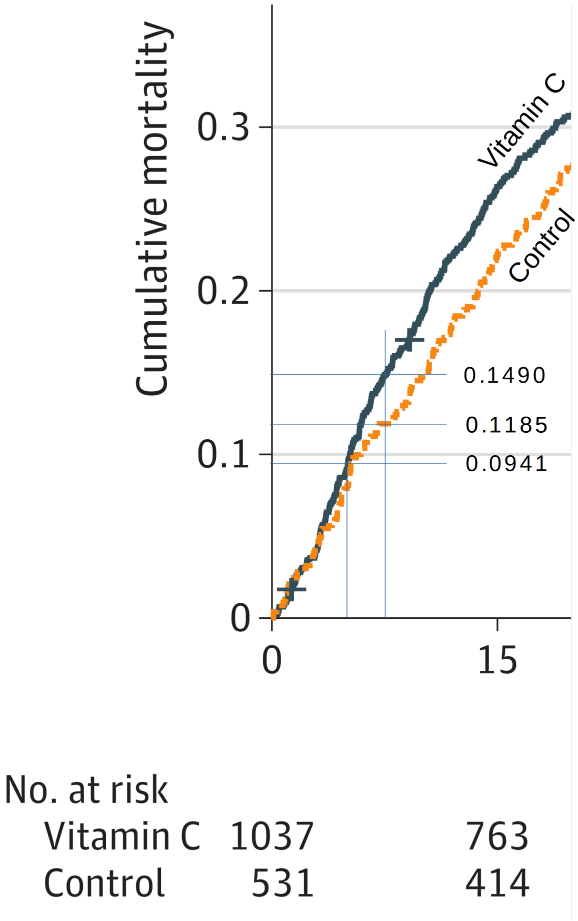

	Vitamin C	Control
Time 0	1037	531
	0.1490	0.1185
At 7.5 days	**154.5**	**62.9**
**Rounded to**	**154**	**63**
**At risk at day 5**	939	481
**Died between day 5 and day 7.5**	**56**	**13**
Alive at day 7.5	883	468
Rate of death between day 5 and 7.5	0.060	0.027
**RR for death between days 5 and 7.5**	**2.207**	

Calculation of the RR to compare vitamin C and control groups

Time range 5 to 15 days (compare page 2)
Printout of the R program

```
riskratio.wald(COVIDd15)
$data
          Outcome
Predictor  Disease1 Disease2 Total
  Exposed1      414       67   481
  Exposed2      763      176   939
  Total        1177      243  1420

$measure
          risk ratio with 95% C.I.
Predictor  estimate lower upper
  Exposed1     1.00    NA    NA
  Exposed2     1.35  1.04  1.74

$p.value
          two-sided
Predictor  midp.exact fisher.exact chi.square
  Exposed1         NA           NA         NA
  Exposed2     0.0215       0.0254     0.0226

$correction
[1] FALSE

attr(,"method")
[1] "Unconditional MLE & normal approximation (Wald) CI"
```

Time range 5 to 7.5 days (compare page 4)
Printout of the R program

```
riskratio.wald(COVIDd7.5)
$data
          Outcome
Predictor  Disease1 Disease2 Total
  Exposed1      468       13   481
  Exposed2      883       56   939
  Total        1351       69  1420

$measure
          risk ratio with 95% C.I.
Predictor  estimate lower upper
  Exposed2     2.21  1.22  3.99

$p.value
          two-sided
Predictor  midp.exact fisher.exact chi.square
  Exposed1         NA           NA         NA
  Exposed2    0.00525      0.00602    0.00683

$correction
[1] FALSE

attr(,"method")
[1] "Unconditional MLE & normal approximation (Wald) CI"
```

Time range 15 to 90 days of Figure 2C [17]
Printout of the R program

```
> #Deads after 15 days
> #Control
> (414-329)
[1] 85
> #vit C
> (763-617)
[1] 146
>
> COVIDd90<- matrix(c(329,617, 85,146),nrow=2)
> riskratio.wald(COVIDd90)
$data
          Outcome
Predictor  Disease1 Disease2 Total
  Exposed1      329       85   414
  Exposed2      617      146   763
  Total         946      231  1177

$measure
          risk ratio with 95% C.I.
Predictor  estimate lower upper
  Exposed1    1.000    NA    NA
  Exposed2    0.932 0.734  1.18

$p.value
          two-sided
Predictor  midp.exact fisher.exact chi.square
  Exposed1         NA           NA         NA
  Exposed2      0.564        0.591      0.565
```

Improvement in Poisson regression by adding vitamin C effect on day range 5.0 to 7.5 days.

Fig. A indicates that the divergence between the vitamin C and control curves starts at about 5.0 days.
Fig. C indicates that the divergence between the vitamin C and control curves ends at about 7.5 days.

Here we calculate the improvement in the Poisson regression model when an independent vitamin C effect is allowed for the time range 5.0 to 7.5 days.

period1 indicates time before 5.0 days, with N=531 (control) and N=1037 (vitC), see Fig. C.
period2 indicates time range 5.0 to 7.5 days, with N=481 (control) and N= 939 (vitC), see Fig. C.
period3 indicates time range 7.5 to 90 days, with N=468 (control) and N= 883 (vitC), see Fig. C.

pdays is the product of persons at the baseline and the days of followup.
For example, on period 2, the pdays is 481 × 2.5 days = 1202.5 pdays in the control group (vitC=0).

deaths during the three time periods were estimated in Figs. A and C.

```
> Adhikari2023
  vitC period2 period3 deaths days persons   pdays
1    0       0       0     50  5.0     531  2655.0
2    0       1       0     13  2.5     481  1202.5
3    0       0       1    139 82.5     468 38610.0
4    1       0       0     98  5.0    1037  5185.0
5    1       1       0     56  2.5     939  2347.5
6    1       0       1    266 82.5     883 72847.5
```

1. The Poisson model that assumes a uniform vitamin C effect over the 90 days.

```
> summary(covid1)

Call:
glm(formula = deaths ~ offset(log(pdays)) + vitC + period2 + 
    period3, family = poisson(), data = Adhikari2023)

Coefficients:
            Estimate Std. Error z value Pr(>|z|)    
(Intercept) -4.02693    0.10074 -39.973   <2e-16 ***
vitC         0.08519    0.08563   0.995    0.320    
period2      0.02919    0.14577   0.200    0.841    
period3     -1.64708    0.09605 -17.147   <2e-16 ***

AIC: 51.365
```

2. The model that allows an independent vitamin C effect for the time period 5.0 to 7.5 days.

Here we test whether the vitamin C effect is different in period2:
we add the interaction term between period2 and the vitamin C effect.

```
> summary(covid2)

Call:
glm(formula = deaths ~ offset(log(pdays)) + vitC * period2 +
    period3, family = poisson(), data = Adhikari2023)

Deviance Residuals:
       1         2         3         4         5         6
 0.03627   0.00000  -0.02170  -0.02584   0.00000   0.01570

Coefficients:
             Estimate Std. Error z value Pr(>|z|)
(Intercept)  -3.97731    0.10149 -39.190   <2e-16 ***
vitC          0.01136    0.08966   0.127   0.8992
period2      -0.54990    0.29534  -1.862   0.0626 .
period3      -1.64764    0.09605 -17.153   <2e-16 ***
vitC:period2  0.78009    0.32065   2.433   0.0150 *

AIC: 46.658
```

Addition of the different vitamin C effect to the second period improves the statistical model by $\chi^2 = 6.7$ which corresponds to **P = 0.0096**.

```
lrtest(covid1,covid2)
Likelihood ratio test

Model 1: deaths ~ offset(log(pdays)) + vitC + period2 + period3
Model 2: deaths ~ offset(log(pdays)) + vitC * period2 + period3
  #Df  LogLik Df  Chisq Pr(>Chisq)
1   4 -21.682
2   5 -18.329  1 6.7069   0.009604 **
```

As expected, the vitamin C effect in period2 is very close to the RR = 2.21 calculated on page 6:

```
> exp(0.78009)
[1] 2.181669
```

3. The model that allows a different vitamin C effect for each of the three time periods.

Finally, we test whether the vitamin C effect is different between periods 1 and 3:
we further add the interaction term between period3 and the vitamin C effect.

```
> summary(covid3)

Call:
glm(formula = deaths ~ offset(log(pdays)) + vitC * period2 +
    vitC * period3, family = poisson(), data = Adhikari2023)


Coefficients:
              Estimate Std. Error z value Pr(>|z|)
(Intercept)  -3.972177   0.141421 -28.088   <2e-16 ***
vitC          0.003619   0.173793   0.021   0.9834
period2      -0.555032   0.311325  -1.783   0.0746 .
period3      -1.654616   0.164907 -10.034   <2e-16 ***
vitC:period2  0.787835   0.353531   2.228   0.0258 *
vitC:period3  0.010546   0.202874   0.052   0.9585

AIC: 48.655
```

Addition of the different vitamin C effect to the third time period does not improve the statistical model at all. Change in χ^2 corresponds to P = 0.96.

```
> lrtest(covid2,covid3)
Likelihood ratio test

Model 1: deaths ~ offset(log(pdays)) + vitC * period2 + period3
Model 2: deaths ~ offset(log(pdays)) + vitC * period2 + vitC *
period3
  #Df  LogLik Df  Chisq Pr(>Chisq)
1   5 -18.329
2   6 -18.328  1 0.0027     0.9586
```

Allowing separate vitamin C effects for each of the three periods makes the period2 vitamin C effect even closer to the RR = 2.21 calculated on page 6.

```
> exp(0.78783)
[1] 2.199
```
